# Supplementary material for: Spatial disinfection potential of slightly acidic electrolyzed water
Source: PLoS One. 2021 Jul 2;16(7):e0253595. doi: 10.1371/journal.pone.0253595 (PMC8253431; doi:10.1371/journal.pone.0253595)
Supplement: Attachment — Submitted filename: PlosOne_FRD_Jan21.docx [file pone.0253595.s002.docx]

| Section | Comment |
| --- | --- |
| Tittle: Spatial disinfection potential of Slightly acidic electrolyzed water |  |
| Abstract |  |
| Introduction |  |
| Materials and methods | |
| L101 – 102 …. on nutrient agar (Eiken Chemical Co.) and *B. subtilis* was cultured on soybean-casein digest agar (Eiken Chemical…  L111. … was cultured on potato dextrose agar….  L118 ….. cultured in a heart infusion broth…  L133… soybean-casein digest medium  L152 – 153 ….ultrasonic humidifier.. | Please add the code number of all used media and machine/equipment not only the brand or which company they are form. |
| L 126 – 128 .. SAEW used in the experiment involving pure cultures had an available chlorine concentration of 30 mg/kg and a pH of 5.9. SAEW used in the spatial disinfection experiment had an available chlorine concentration of 41 mg/kg and a pH of 6.0. | Why the available chlorine concentration used for pure culture and in spatial disinfection experiment were different? |
| 2.3. Effects of SAEW on pure cultures | How many replication samples you’ve done for each microorganism? |
| 2.4. Effects of indoor spraying of SAEW | How many replications process (not sampling time) you’ve done in this study? |
| Statistical analysis | Please provide information about the statistical tool that been used. |
| Discussion | |
|  | The discussions are not deep enough. I can’t see the discussion of the mechanism on how the SAEW can inhibit or reduce the microbial growth in the studies. |
| General comments | - The manuscript is well written. However, some details are missing. - It is crucial to give the detail of all medias and used equipment/tools/machines being used. This information is needed to verify your methods and enable the reader mimicking your study. - The discussions are not deep enough. I can’t see the discussion of the mechanism on how the SAEW can inhibit or reduce the microbial growth in the studies. |
