# Supplementary material for: Spatial disinfection potential of slightly acidic electrolyzed water
Source: PLoS One. 2021 Jul 2;16(7):e0253595. doi: 10.1371/journal.pone.0253595 (PMC8253431; doi:10.1371/journal.pone.0253595)
Supplement: Attachment — Submitted filename: Response to Reviewers.docx [file pone.0253595.s003.docx]

March 25, 2021

Dear Editor-in-Chief and Reviewers of PLOS ONE,

We would like to thank the editor and the reviewers for their time to read our manuscript PONE-D-20-33738 “Spatial disinfection potential of slightly acidic electrolyzed water” and giving us valuable comments and suggestions to improve its quality. We have revised the manuscript accordingly. The sections that we modified are highlighted in yellow in the manuscript. Our responses to the editor and reviewers’ comments are in the following pages.

We hope that our revised manuscript will be suitable for publication in PLOS ONE.

Yours faithfully,

Angelica Naka

Project Researcher

Graduate School of Agricultural and Life Sciences

The University of Tokyo

1-1-1 Yayoi, Bunkyo-ku, Tokyo 113-0032

Agriculture Building 6, office 104-1, Japan

Tel: +81-3-5841-7535　Fax: +81-3-5841-8041

E-mail: angelica.naka@mail.ecc.u-tokyo.ac.jp

| Comment | Comments and suggestions | Answer |
| --- | --- | --- |
| 1.1 | In addition to review comments, you failed to disclose any potential conflict of interest with the SAEW solution used your study. Please ensure to include the product number of the SAEW used, if it is commercially available, otherwise, provide details pertaining to SAEW formulation. Funding details are also not disclosed, please add this information in the acknowledgment section. | We would like to thank the editor for the valuable comments and suggestions.  We conducted this research with the annual budget of the University of Tokyo. However, this budget was to conduct several research projects, not specifically to work on SAEW research. Following the editor's advice (by email), we left the Acknowledgements section unchanged:  Lines 309-310  “The authors would also like to thank the anonymous reviewer for their valuable comments.”  We discussed experimental design and results (conceptualization and validation) with Organo Food Tech Corporation. They also provided the SAEW and the clean chamber for the spatial disinfection test (resources). Regarding SAEW, we added the following information:  Lines 128-129  “Slightly acidic electrolyzed water (HOCL0.36 t, HOCL Inc.) was specifically prepared by Organo Food Tech, Saitama, Japan for the purpose of these experiments.” |
| 1.2 | When reviewing the experimental design dealing with indoor spraying of SAEW, no information was provided as to why *S. epidermidis* was the only selected organism for this study. Why were other previously investigated species not tested in this assay, considering that outcomes may vary from the use of SAEW directly on pure culture or indirectly via indoor spraying? | We conducted spatial disinfection experiments only with *S. epidermidis* because it is generally regarded as a safe microorganism that is present in human skin and usually does not cause pyogenic infections unless there are compromised patients. One of the objectives of this research is to report on the efficacy of SAEW when used as spray (mist). Considering that we do not have a proper infrastructure to spray pathogenic and infectious microorganisms, we selected *S. epidermidis* for spatial disinfection experiments. We added the following sentence:  Lines 122-125  “*S. epidermidis* was the only microorganism selected for spatial disinfection experiments because it is a common bacterium present in the human skin and, thus, does not usually cause infections in non-compromised patients and does not require high level of biosafety laboratories.”  To show the effectiveness of SAEW on other pathogenic bacteria, we conducted experiments on pure culture. |
| 1.3 | Finally, the antimicrobial potency of SAEW in your study was established based on CFU counts. Have you looked into other methods for validating cell death? | No, we have not investigated other methods for validating cell death. We used CFU count method because we found that this is one of the most common methods used by research teams that worked on research related to bacterium removal efficacy. |
| 2.1 | The authors submitted a document about the use of slightly acidic electrolyzed water on spatial disinfection. It is a very relevant document however, there are some points that need to be clarified.  L42. Authors stablished that sodium hypochlorite is highly safe however in some European countries, its use is banned. It will need to be modify. | The authors would like to thank the reviewer for the feedback and suggestions to improve our manuscript.  The reviewer is right, even though the use of sodium hypochlorite is still allowed in some countries such as Japan and Korea, its use is banned in many countries. So, we modified the sentence as follows:  Lines 42-44  “Sodium hypochlorite solutions are inexpensive and relatively effective, so they have been used around the world for over 100 years.” |
| 2.2 | L74. The manuscript stablished: “Spatial disinfection with a conventional agent involves spraying in an unoccupied space or using protective equipment. Spatial disinfection can be performed with a low concentration of SAEW” At the end of the paragraph, authors cited this document https://doi.org/10.1637/11107-042115-Reg.1where it is stablished that Newcastle virus is affected by the use of higher concentration of SAEW (100 ppm). Authors need to clarify its statement. | The reviewer is right, Newcastle virus was affected by 50 and 100 mg/L of free chlorine concentration. SAEW disinfection effectiveness depends on the type and concentration of the microorganisms. It also depends on the SAEW’s free chlorine concentration and the sprayed volume and duration. Unfortunately, there are very few experiments on spatial disinfection effectivity of SAEW. We added references of research works that were conducted with 30 mg/L and 80 mg/L of free chlorine concentration. Please refer to the following paragraph:  Lines 77-82  “Spatial disinfection can be performed spraying SAEW with free chlorine concentrations of 100 mg/L or lower and without concern for residual chlorine. There are numerous studies on the sterilizing action of electrolyzed water, but there are very few studies on the use of SAEW for indoor disinfection [4-6].” |
| 2.3 | L80. It is a little confuse because authors wrote about the impact of multidrug-resistant microorganisms and the necessity for big area disinfection like food factories, hospitals,  care facilities, kindergartens, and animal pens and coops. So, it is implying that the study will focus about airborne pathogens. But, at L94, most of the pathogens are not airborne. I believe authors needs to justify it statement why they are using bacteria like Salmonella enterica, Staphylococcus aureus and/or E. coli. | The reviewer is correct, not all species tested in this research are airborne bacteria.  One of the objectives of this research is to evaluate the efficacy of SAEW against bacteria when applied as spray (mist). Unfortunately, our laboratory does not have a proper infrastructure to spray pathogenic and infectious bacteria. So, we conducted spray experiments only with *S. epidermidis* which is generally regarded as a safe microorganism unless there are compromised patients. To evaluate the effectiveness of SAEW against pathogenic bacteria and bacterial spores, we applied SAEW directly to pure culture (we can handle them in our biological safety cabinet).  We added the following paragraph:  Lines 77-82  “Its efficacy as disinfectant was measured in this research by directly applying SAEW on different types of bacteria and bacterial spores. Spatial disinfection can be performed spraying SAEW with free chlorine concentrations of 100 mg/L or lower and without concern for residual chlorine. There are numerous studies on the sterilizing action of electrolyzed water, but there are very few studies on the use of SAEW for indoor disinfection [4-6].” |
| 2.4 | L172. It is confused when in text authors use CFU/mL, then on L174 log10 CFU/mL. Please, use only one type of units. I recommend using log10 CFU/mL. | The reviewer is right. We corrected the mistake as follows:  Lines 176-177  “Treatment of B. cereus (spores), a gram-positive bacillus that causes food to spoil, resulted in 1/10 of the original log_10_ CFU/mL after 60 sec.” |
| 2.5 | L276. There is no reference on the use of EW on those facilities. | Following the reviewer’s suggestion, we added the references accordingly.  Lines 288-290  “SAEW has been sprayed at numerous sites such as hospitals, care facilities, kindergartens, and greenhouses in Japan over the past few years [8, 22], with no reports of health incidents due to the spraying.” |
| 2.6 | Authors worked with *S. epidermidis*, which causes biofilms. Results are interesting however, I suggest performing an in vitro study with this bacteria and to sample walls of the chamber where indoor study was performed. This will provide extra information about the survival/escaped bacteria after EW air treatment.  Because SAEW is not a neutral water, its pH could affect/damage different surfaces. It could be interesting to perform a corrosion test. | We performed two type of experiments:   - In vitro experiments with pathogenic and infectious bacteria and bacterial spores. - Spray experiment with *S. epidermidis*, regarded as a safe microorganism (it is present in the human skin) and usually does not cause pyogenic infections unless there are compromised patients.   The reviewer is correct, some bacteria may adhere to the walls. However, considering that SAEW’s free chlorine concentration is 41 ppm (this means that 99.59% is water), we believe that SAEW spray is not suitable for surface disinfection. For surface disinfection, it is more effective if we apply SAEW directly and in great amount. In this experiment we focused on bacteria that were suspended in the chamber and tested both SAEW and water, the latter as control. Hypochlorous acid (HOCl), which is the active molecule in SAEW, easily reacts with organic matter. Thus, if applied as spray (considering that HOCl concentration in SAEW is low), it will probably react with some dust on the surface and not with the bacteria.  Regarding experiments with slightly acidic water, it could be interesting, but we believe that pH 6 may not have great impact on bacteria tested in this research. These bacteria usually show protection mechanisms against acid stress and overcome the challenge posed by different acidic environments. |
| 3.1 | L101 – 102 …. on nutrient agar (Eiken Chemical Co.) and B. subtilis was cultured on soybean-casein digest agar (Eiken Chemical…  L111. … was cultured on potato dextrose agar….  L118 ….. cultured in a heart infusion broth…  L133… soybean-casein digest medium  L152 – 153 ….ultrasonic humidifier..  Please add the code number of all used media and machine/equipment not only the brand or which company they are form. | The reviewer is correct. We followed the reviewer suggestion and added the necessary information.  Lines 101-102  “*B. cereus* was cultured on nutrient agar PT2810 (Eiken Chemical Co.) and *B. subtilis* was cultured on soybean-casein digest agar PT8010 (Eiken Chemical Co.)”  Lines 108-109  “*E. coli, P. aeruginosa, S. enterica, and S. aureus* were each cultured on nutrient agar PT 1010 (Eiken Chemical Co.) at 35℃±1℃ for 18–24 h.”  Lines 111-113  “*C. cladosporioides* was cultured on potato dextrose agar PT 2610 (Eiken Chemical Co.) at 25℃±1℃ for 7–10 days.”  Lines 118-120  “The test bacterium was cultured in a brain heart infusion broth PT3025 (Eiken Chemical Co.) at 35℃±1℃ for 18–24 h.”  Lines 136-145  “This test solution was stored at 20℃±1℃. After 15 s, 1 min, and 15 min, it was immediately diluted 10-fold with soybean-casein digest medium with polysorbate 80 & lecithin SCDLP “DAIGO” (Nihon Pharmaceutical Co.). Microorganisms in the test solution were counted using a medium for cultivation and isolation. The medium for cultivation and isolation was soybean-casein digest agar with polysorbate 80 & lecithin SCDLP “DAIGO” (Nihon Pharmaceutical Co.) for *B. cereus, B. subtilis, E. coli, P. aeruginosa, S. enterica, and S. aureus*. Each bacterium was then cultured at 35℃±1℃ for 2 days using the pour plate method. The medium for cultivation and isolation was glucose peptone agar with lecithin & polysorbate 80 GPLP AGAR “DAIGO” (Nihon Pharmaceutical Co.) for *C. cladosporioides*.”  Lines 155-158  “Three mL of this mixture (2 mL of the bacterial solution + 1 mL of PBS) was sprayed at a rate of 1 mL/min for 3 min from the spray nozzle of an ultrasonic humidifier AHD-012 (Shizuku Plus+, Apix International, Osaka, Japan) in a large clean chamber.” |
| 3.2 | L 126 – 128 .. SAEW used in the experiment involving pure cultures had an available chlorine concentration of 30 mg/kg and a pH of 5.9. SAEW used in the spatial disinfection experiment had an available chlorine concentration of 41 mg/kg and a pH of 6.0.  Why the available chlorine concentration used for pure culture and in spatial disinfection experiment were different? | We conducted spatial disinfection experiments as soon as we received the SAEW from Organo Food Tech Corporation. At that time, the concentration of free chlorine was 41 mg/L. Some weeks later, we conducted experiments on pure culture. At that time, the concentration was 30 mg/L (free chlorine concentration decreased with time, but it was still effective). We measured free chlorine concentration just before experiments. |
| 3.3 | 2.3. Effects of SAEW on pure cultures  How many replication samples you’ve done for each microorganism? | We performed experiments in triplicate. We added this information in the manuscript. Please refer to Line 148.  Line 148  “All experiments were performed in triplicate.” |
| 3.4 | 2.4. Effects of indoor spraying of SAEW    How many replications process (not sampling time) you’ve done in this study? | We performed indoor spraying experiments three times. We added this information in the manuscript. Please refer to Line 163.  Line 163  “This experiment was repeated three times.” |
| 3.5 | Statistical analysis  Please provide information about the statistical tool that been used. | The obtained results were statistically assessed using Tukey’s honestly significant difference (HSD) test. Please refer to Lines 167-169.  Lines 167-169  “The obtained results were statistically assessed using Tukey’s honestly significant difference (HSD) test, with P < 0.05 considered to be statistically significant.” |
| 3.6 | Discussion    The discussions are not deep enough. I can’t see the discussion of the mechanism on how the SAEW can inhibit or reduce the microbial growth in the studies. | The reviewer is right. We added the following paragraph regarding SAEW’s bacteria inhibition mechanism.  Lines 278-286  “There are few studies on the disinfection mechanism of SAEW. Ding et al. [19] found that SAEW disrupted permeability of the cell membrane and the cytoplasmic ultrastructure in S. aureus cells. Kim at al. [20] studied cell morphological states and cell permeability through a transmission electron microscope and found that SAEW could penetrate bacterial cell walls and induce cell damage and disruption. Wigginton el al. [21] identified the virus inactivation mechanisms as free chlorine (FC), reporting that FC caused losses in both genome replication and protein-mediated functions. They suggested that FC altered the capsid structure which facilitates access to protein structures that are inside.” |
| 3.7 | General comments  - The manuscript is well written. However, some details are missing.  - It is crucial to give the detail of all medias and used equipment/tools/machines being used. This information is needed to verify your methods and enable the reader mimicking your study.  - The discussions are not deep enough. I can’t see the discussion of the mechanism on how the SAEW can inhibit or reduce the microbial growth in the studies. | We would like to thank the reviewer for the feedback. We revised the manuscript according to the reviewer’s comments and suggestions. |
