# Supplementary material for: Spatial disinfection potential of slightly acidic electrolyzed water
Source: PLoS One. 2021 Jul 2;16(7):e0253595. doi: 10.1371/journal.pone.0253595 (PMC8253431; doi:10.1371/journal.pone.0253595)
Supplement: Attachment — Submitted filename: Response to Reviewers June 3.docx [file pone.0253595.s004.docx]

June 3, 2021

Dear Editor-in-Chief and Reviewers of PLOS ONE,

We would like to thank the editor and the reviewers for their time to read our manuscript PONE-D-20-33738R1 “Spatial disinfection potential of slightly acidic electrolyzed water” and giving us valuable comments and suggestions to improve its quality. We have revised the manuscript accordingly. The sections that we modified are highlighted in yellow in the manuscript. Our responses to the editor and reviewers’ comments are in the following pages.

We hope that our revised manuscript will be suitable for publication in PLOS ONE.

Yours faithfully,

Angelica Naka

Project Researcher

Graduate School of Agricultural and Life Sciences

The University of Tokyo

1-1-1 Yayoi, Bunkyo-ku, Tokyo 113-0032

Agriculture Building 6, office 104-1, Japan

Tel: +81-3-5841-7535　Fax: +81-3-5841-8041

E-mail: angelica.naka@mail.ecc.u-tokyo.ac.jp

| Comment | Comments and suggestions | Answer |
| --- | --- | --- |
| 1.1 | I previously suggested to authors to perform an in vitro study with S. epidermidis, authors answer me back the following statement: we believe that pH 6 may not have great impact on bacteria tested in this research. These bacteria usually show protection mechanisms against acid stress and overcome the challenge posed by different acidic environments.  It is not clear why authors make that statement if results show that spatial disinfection worked properly. I suggest including an in vitro study with S. epidermidis. | We would like thank the reviewer for the valuable comment and suggestion.  As suggested by the reviewer, we performed in vitro experiments with *S. epidermidis* and added the results in the manuscript. Please refer to Lines 94-100, 113-116, 144-146, 187-189, 193-196 and 309-310 and Figure 1.  Lines 94-100  An in vitro experiment was performed with *Bacillus cereus* NBRC 13494, *Bacillus subtilis* NBRC 3134, *Pseudomonas aeruginosa* NBRC 13275, *Salmonella enterica subsp. enterica* NBRC 3313, *Staphylococcus aureus subsp. aureus* NBRC 12732, *Cladosporium cladosporioides* NBRC 6348, and *Staphylococcus epidermidis* NBRC 12993 from the NITE Biological Resource Center (NBRC, Japan) and *Escherichia coli* ATCC 43895 (serotype O157:H7, a verotoxin I and II-producing strain) from the American Type Culture Collection (ATCC, USA).  Lines 113-116  *S.* *epidermidis* was cultured in a brain heart infusion broth PT3025 (Eiken Chemical Co.) at 35℃±1℃ for 18–24 h. The bacterial count was adjusted to 10^7^-10^8^ CFU/mL with a sterile phosphate buffer (phosphate-buffered saline, or PBS) to yield the test bacterial solution.  Lines 144-146  The medium for cultivation and isolation was soybean-casein digest agar with polysorbate 80 & lecithin SCDLP “DAIGO” (Nihon Pharmaceutical Co.) for *B. cereus, B. subtilis, E. coli, P. aeruginosa, S. enterica, S. aureus*, and *S. epidermidis*  Lines 187-189  Treatment of *S. aureus* and *S. epidermidis*, gram-positive facultative anaerobic bacilli, with electrolyzed water resulted in logarithmic (log_10_ CFU/mL) decrease of 5.2 or greater and 5.4 or greater, respectively within 15 sec.  Lines 193-196  Fig 1. Effects of SAEW on pure cultures (a) *Bacillus cereus*, (b) *Bacillus subtilis*, (c) *Escherichia coli* (O157:H7), (d) *Pseudomonas aeruginosa*, (e) *Salmonella enterica subsp. enterica*, (f) *Staphylococcus aureus subsp. aureus*, (g) *Cladosporium cladosporioides*, and (h) *Staphylococcus epidermidis*.  Line 307-310  Results indicated that SAEW with an available chlorine concentration of about 30 mg/L has sufficient sterilizing action, even able to kill *Bacillus* spores within 15 minutes. *E. coli*, *P aeruginosa*, *S. enterica*, *S. aureus*, and *S. epidermidis* were eliminated within 15 sec of 30 mg/L of SAEW exposure.  As for the statement regarding pH, the reviewer is correct, this does not answer the reviewer’s question. We wanted to mention that the disinfectant capacity of SAEW is attributed to the presence of HOCl. |
